# Optimal Extent of Lymph Node Dissection for Non‐Metastatic Colon Cancer by Tumor Location: Evaluation of the Therapeutic Value Index for Each Lymph Node Station

**DOI:** 10.1002/ags3.70023

**Published:** 2025-04-21

**Authors:** Akira Ouchi, Kozo Kataoka, Eiji Shinto, Takashi Akiyoshi, Takefumi Yoshida, Yasuyuki Takamizawa, Yukihide Kanemitsu, Hirotoshi Kobayashi, Yoichi Ajioka, the Lymph Nodes Committee of JSCCR

**Affiliations:** ^1^ Department of Gastroenterological Surgery Aichi Cancer Center Hospital Nagoya Aichi Japan; ^2^ Division of Lower GI, Department of Gastroenterological Surgery Hyogo Medical University Nishinomiya Hyogo Japan; ^3^ Department of Surgery National Defense Medical College Saitama Japan; ^4^ Department of Gastroenterological Surgery, Cancer Institute Hospital Japanese Foundation for Cancer Research Tokyo Japan; ^5^ Department of Surgery Kurume University School of Medicine Fukuoka Japan; ^6^ Department of Colorectal Surgery National Cancer Center Hospital Tokyo Japan; ^7^ Department of Surgery, Mizonokuchi Hospital Teikyo University Kawasaki Kanagawa Japan; ^8^ Division of Molecular and Diagnostic Pathology, Graduate School of Medical and Dental Sciences Niigata University Niigata Japan

**Keywords:** colonic neoplasms, D3 dissection, lymph node excision, N3 classification, therapeutic value index

## Abstract

**Aims:**

To determine the optimal extent of lymph node dissection for non‐metastatic colon cancer by tumor location based on the therapeutic value index (TVI) for each lymph node station.

**Methods:**

Consecutive patients with surgical stage I–III colon or rectosigmoid cancer in the Japanese Society for Cancer of the Colon and Rectum database who underwent curative resection between January 2003 and December 2014 were analyzed. The TVI was defined as the incidence of lymph node metastasis multiplied by 5‐year overall survival and calculated for each nodal station stratified by tumor location.

**Results:**

A total of 33 231 patients were eligible for analysis. In cecal cancer, the TVI was 2.086 for nodal station #203, but only 0.000 for #213. In ascending colon cancer, the TVI was 1.080 for #203 and 0.644 for #213, but only 0.178 for #223. In transverse colon cancer, the TVI was 1.942 for #223, but only 0.066 for #213 and 0.159 for #203. In descending colon cancer, the TVI was 0.215 for #253. The TVI was 1.172 for #253 in sigmoid colon cancer and 1.155 for #253 in rectosigmoid cancer.

**Conclusion:**

Considering that a previous systematic review reported TVIs in the range of 0.295–0.576 for the para‐aortic lymph nodes in patients with colorectal cancer, dissection of the main lymph nodes along the feeding artery has a therapeutic value in non‐metastatic colon cancers. Meanwhile, the significance of #253 dissection for descending colon cancer requires further discussion.

## Graphical abstract

Lymph node dissection of the main nodes along the feeding artery has a therapeutic value in early‐stage colon cancers. Meanwhile, the significance of #253 dissection for descending colon cancer requires further discussion.

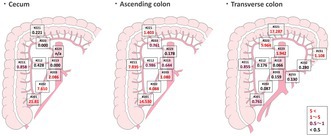

## 1 Introduction

The primary treatment for non‐metastatic colon cancer is curative surgical resection [1, 2]. Although there is some variation in the surgical procedures performed from country to country, it is broadly accepted that the oncologic principles for colon cancer surgery include resection of the tumor‐containing segment of the colon along with its draining lymph node basin [3].

The Japanese Society for Cancer of the Colon and Rectum (JSCCR) recommends D2/D3 dissection as one of the procedures for oncologic resection of colon cancer. The principles underlying this recommendation include sharp excision of the mesocolic plane, the optimal margin of bowel resection, and sufficient lymph node dissection by tumor stage based on the anatomy of the lymphatic system [4, 5]. D2/D3 dissection, which is standardized in Japan, corresponds to the complete mesocolic excision with central vascular ligation performed in Europe [6, 7, 8]. Specimens resected by D2/D3 dissection and complete mesocolic excision with central vascular ligation have been reported to be pathologically equivalent, and the oncologic outcomes of D2/D3 dissection are satisfactory and comparable with Western case series [9, 10]. For example, the JCOG2003A study, an integrated analysis of a Japanese cohort, found 5‐year overall survival (OS) rates of 97.2%–96.0% in patients with American Joint Committee on Cancer (AJCC) stage II colon cancer and 87.6%–90.7% in those with AJCC stage III colon cancer [11], which were superior to the data published by the US National Cancer Database [12].

Meanwhile, although each D2 and D3 lymph node dissection has been defined as “complete pericolic/perirectal and intermediate lymph node dissection” and “pericolic/perirectal, intermediate, and main lymph nodes” in the JSCCR classification [13], the optimal extent of lymph node dissection by tumor location is still unclear. In particular, the relatively low incidence of main lymph node metastasis (LNM) has made it difficult to investigate the optimal extent of main lymph node dissection along the superior mesenteric and inferior mesenteric arteries.

The JSCCR Lymph Nodes Committee launched a task force for “re‐definition of N3 classification” in 2023, initiating a project to optimize the extent and indications for D3 dissection in colon cancer by evaluating the therapeutic value index (TVI) for each lymph node station. This project involved a two‐stage analysis—first by tumor location and then by tumor stage. Our initial focus was to clarify the optimal extent of D3 lymph node dissection for non‐metastatic colon cancers, calculating TVI by tumor location.

## 2 Methods

### 2.1 Data Sources

This study analyzed data from the JSCCR colorectal cancer (CRC) registration system (JSCCR database), which is an original survey research program for CRC in Japan [14]. The JSCCR database was started in 1980, includes data for over 200, 000 patients treated for CRC in Japan between 1974 and 2014, and is now registering over 13, 000 patients annually from over 80 dedicated institutions for CRC across Japan. All institutions participating in the JSCCR database follow the guidelines of the JSCCR, including the indication of D3 dissection, for the treatment of non‐metastatic CRC.

**FIGURE 1:**
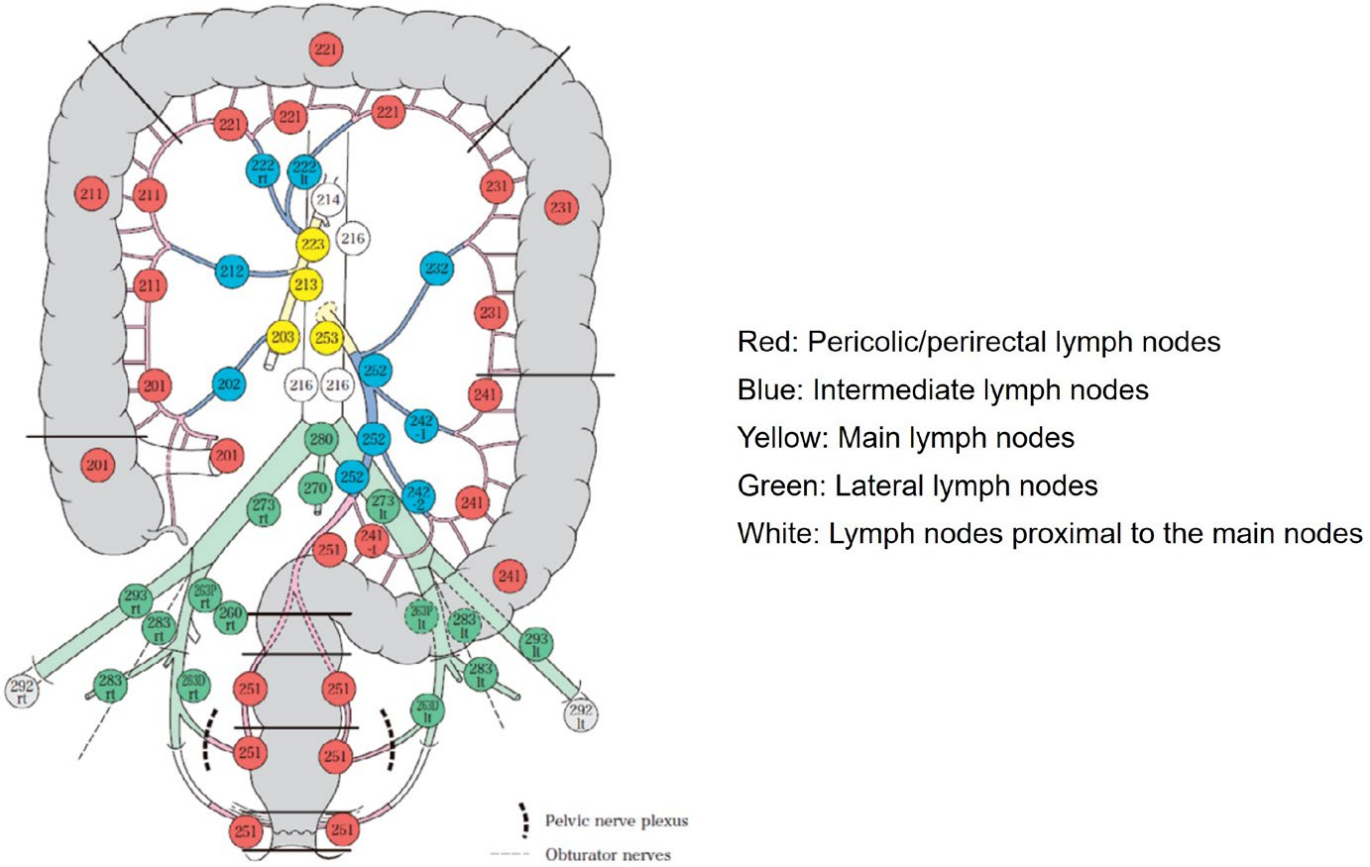
Lymph node groups and station numbers according to the JSCCR classification. Reprinted with permission from the Japanese Society for Cancer of the Colon and Rectum and Kanehara & Co. Ltd.: Japanese Classification of Colorectal, Appendiceal, and Anal Carcinoma‐ the 3rd English edition, 2019. JSCCR, Japanese Society for Cancer of the Colon and Rectum.

The JSCCR database contains detailed information on lymph node status by nodal group (station) as defined by the JSCCR classification [13] (Figure 1) and also includes the extent of lymph node dissection, which is clearly stated in the JSCCR classification. The presence of main lymph node metastasis is categorized as N3 by the JSCCR classification. D3 dissection is defined as complete dissection of the regional lymph nodes, including the pericolic, intermediate, and main lymph nodes at the root of the supplying artery.

### 2.2 Patient Selection

Consecutive patients with CRC between January 2003 and December 2014 were identified in the JSCCR database. Patient background characteristics were extracted, including demographics, clinical and pathological characteristics, details of the extent of lymph node dissection, lymph node status, and survival.

Patients with JSCCR surgical stage I–III colon or rectosigmoid cancer (cecum to rectosigmoid) who underwent curative resection were included. Patients with lesions other than adenocarcinoma or lesions of unknown pathology, those with cancers other than colon or rectosigmoid (e.g., small bowel, appendiceal, rectal, or perineal skin cancers), those in whom the tumor location was unknown, and those with surgical stage 0/IV/unknown cancer were excluded. Patients without D3 dissection (D0/1/2/unknown), those with non‐curative surgical resection (sR1/2/unknown), those with no information on lymph node status (pNX/station unknown), and those with no survival data available were also excluded to enable accurate assessment of lymph node status and survival.

### 2.3 Primary Outcome Measure

The primary outcome measure was the TVI, which was first proposed by Sasako et al. [15] TVI is a relative measure that can be used to compare survival in patients who have positive nodes and undergo lymph node dissection and estimates the therapeutic effect of lymph node dissection in each station. TVI is defined as the incidence of LNM multiplied by 5‐year OS, and a higher TVI corresponds to a greater therapeutic effect of lymph node dissection in each nodal station.

In this study, patients were stratified by tumor location according to the JSCCR classification, namely, the cecum, ascending colon, transverse colon, descending colon, sigmoid colon, and rectosigmoid. TVI of each nodal station was calculated by tumor location.

### 2.4 Statistical Analysis

Categorical variables were analyzed using Pearson's chi‐squared test. Continuous variables were analyzed using the Mann–Whitney *U* test and are presented as the median (interquartile range). OS was estimated by Kaplan–Meier survival analysis, and TVI was evaluated as described above. All statistical analyses were performed using STATA SE version 18.0 (StataCorp LLC, College Station, TX, USA).

## 3 Results

### 3.1 Patient Characteristics

**FIGURE 2:**
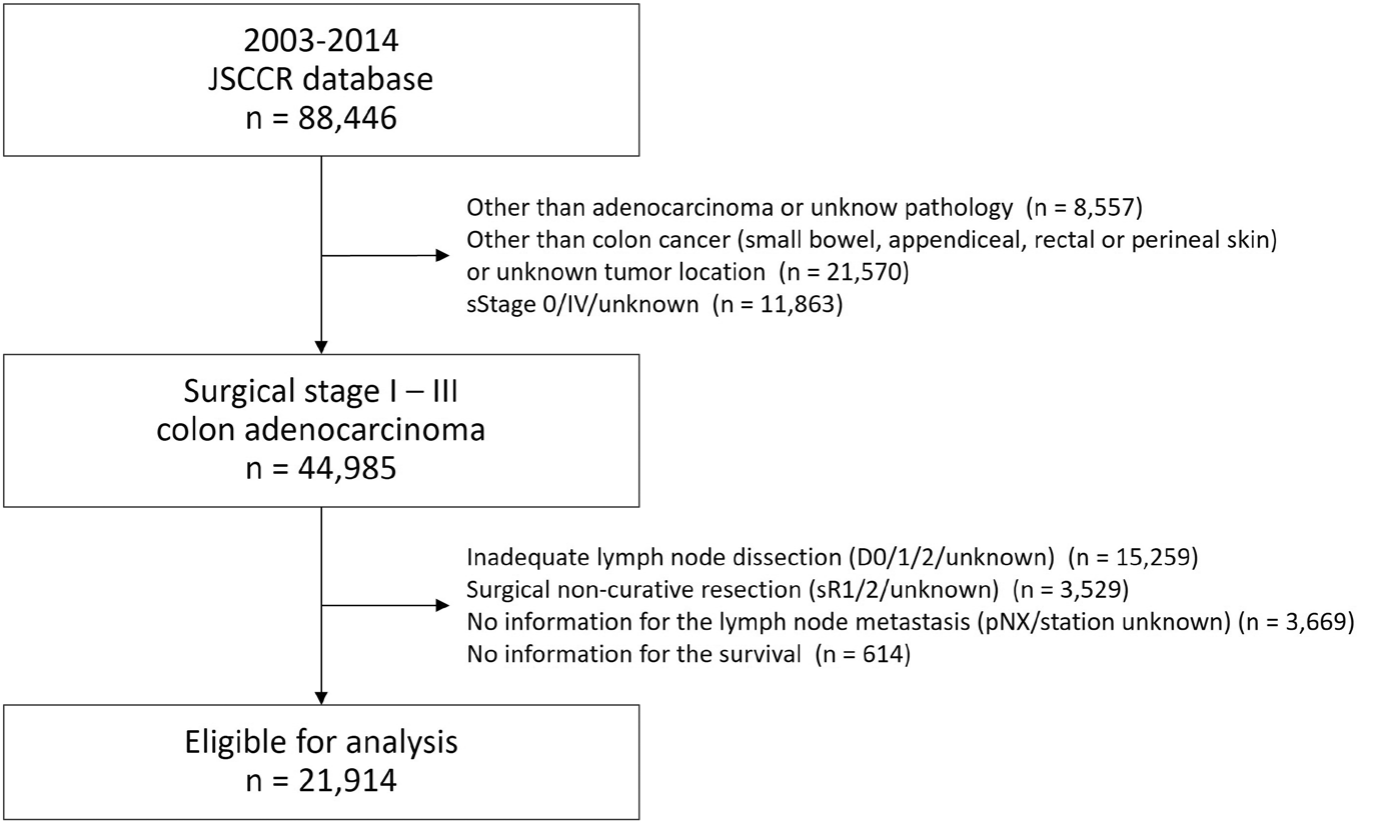
Patient flow diagram. JSCCR, Japanese Society for Cancer of the Colon and Rectum.

In total, 43 461 of the 88 446 patients identified in the JSCCR database were excluded, leaving 44 985 patients with JSCCR surgical stage I–III colon cancer for enrollment in the study (Figure 2). A further 23 071 patients were excluded due to missing lymph node status or survival evaluation, leaving data for 21 914 patients eligible for analysis.

Patient characteristics and tumor locations are shown in Table 1. Patients with cecal or ascending colon cancer included a greater proportion of older and female patients compared with transverse or left‐sided colon cancer. Also, patients with cecal or ascending colon cancer were more frequently surgical node‐positive than those with tumors at other locations. There was a smaller proportion of patients with transverse and descending colon cancers who underwent minimally invasive surgery compared with cecal, ascending colon, sigmoid colon, or rectosigmoid cancers. There was no clear difference in pathological T classification, N classification, margin status, or postoperative treatment according to tumor location.

**TABLE 1:** Patient characteristics.

	All (*n* = 21 914)	Cecum (*n* = 2280)	Ascending colon (*n* = 5073)	Transverse colon (*n* = 2230)	Descending colon (*n* = 1165)	Sigmoid colon (*n* = 7463)	Rectosigmoid colon (*n* = 3703)
Median age, years [IQR]	69 [61, 75]	71 [63, 78]	71 [64, 78]	70 [62, 77]	68 [61, 74]	67 [60, 74]	66 [58, 73]
Sex, *n* (%)							
Male	11 972 (54.63)	1002 (43.95)	2480 (48.89)	1212 (54.35)	704 (60.43)	4317 (57.85)	2257 (60.95)
Female	9934 (45.33)	1278 (56.05)	2590 (51.05)	1017 (45.61)	461 (39.57)	3143 (42.11)	1445 (39.02)
Unknown	8 (0.04)	0 (0.00)	3 (0.06)	1 (0.04)	0 (0.00)	3 (0.04)	1 (0.03)
JSCCR sT classification, *n* (%)							
sT1	1644 (7.50)	204 (8.95)	452 (8.91)	141 (6.32)	70 (6.01)	541 (7.25)	236 (6.37)
sT2	2755 (12.57)	304 (13.33)	676 (13.33)	225 (10.09)	110 (9.44)	929 (12.45)	511 (13.80)
sT3	9905 (45.20)	919 (40.31)	2400 (47.31)	1034 (46.37)	531 (45.58)	3359 (45.01)	1662 (44.88)
sT4a	5558 (25.36)	583 (25.57)	1182 (23.30)	638 (28.61)	335 (28.76)	1841 (24.67)	979 (26.44)
sT4b	2052 (9.36)	270 (11.84)	363 (7.16)	192 (8.61)	119 (10.21)	793 (10.63)	315 (8.51)
JSCCR sN classification, *n* (%)							
sN0	11 477 (52.37)	1105 (48.46)	2530 (49.87)	1167 (52.33)	621 (53.30)	4086 (54.75)	1968 (53.15)
sN+	10 437 (47.63)	1175 (51.54)	2543 (50.13)	1063 (47.67)	544 (46.70)	3377 (45.25)	1735 (46.85)
Surgical margin status, *n* (%)							
sR0	21 914 (100)	2280 (100)	5073 (100)	2230 (100)	1165 (100)	7463 (100)	3703 (100)
Surgical approach, *n* (%)							
Open	12 125 (55.33)	1274 (55.88)	2825 (55.69)	1374 (61.61)	705 (60.52)	3.943 (52.85)	2004 (54.12)
MIS	8911 (40.66)	918 (40.26)	2038 (40.17)	760 (34.08)	424 (36.39)	3248 (43.52)	1823 (41.13)
Unknown	878 (4.01)	88 (3.86)	210 (4.14)	96 (4.30)	36 (3.09)	272 (3.64)	176 (475)
Lymph node dissection, *n* (%)							
D3	21 914 (100.00)	2280 (100.00)	5073 (100.00)	2230 (100.00)	1165 (100.00)	7463 (100.00)	3703 (100.00)
Histology, *n* (%)							
tub1/tub2	20 363 (92.92)	1997 (87.59)	4516 (89.02)	1992 (89.33)	1094 (93.91)	7187 (96.30)	3577 (96.60)
por/muc/sig	1551 (7.08)	283 (12.41)	557 (10.98)	238 (10.67)	71 (6.09)	276 (3.70)	126 (3.40)
JSCCR pT classification, *n* (%)							
pT0	2 (0.01)	0 (0.00)	0 (0.00)	1 (0.04)	0 (0.00)	1 (0.01)	0 (0.00)
pTis	269 (1.23)	63 (2.76)	82 (1.62)	28 (1.26)	15 (1.29)	54 (0.72)	27 (0.73)
pT1	1810 (8.26)	216 (9.47)	475 (9.36)	146 (6.55)	63 (5.41)	616 (8.25)	294 (7.94)
pT2	2884 (13.16)	361 (15.83)	601 (11.85)	232 (10.40)	123 (10.56)	1023 (13.71)	544 (14.69)
pT3	12 007 (54.79)	1097 (48.11)	2896 (57.09)	1264 (56.68)	695 (59.66)	4058 (54.37)	1997 (53.93)
pT4a	3.756 (17.14)	375 (16.45)	811 (15.99)	433 (19.42)	214 (18.37)	1273 (17.06)	650 (17.55)
pT4b	1141 (5.21)	162 (7.11)	197 (3.88)	120 (5.38)	54 (4.64)	425 (5.69)	183 (4.94)
Unknown	45 (0.21)	6 (0.26)	11 (0.22)	6 (0.27)	1 (0.09)	13 (0.17)	8 (0.22)
JSCCR pN classification, *n* (%)							
pN0	14 648 (66.84)	1524 (66.84)	3480 (68.60)	1553 (69.64)	805 (69.10)	4945 (66.26)	2341 (63.22)
pN1	5068 (23.13)	487 (21.36)	1100 (21.68)	466 (20.90)	283 (24.29)	1813 (24.29)	919 (24.82)
pN2	1598 (7.29)	182 (7.98)	334 (6.58)	116 (5.20)	65 (5.58)	553 (7.41)	348 (9.40)
pN3	600 (2.74)	87 (3.82)	159 (3.13)	95 (4.26)	12 (1.03)	152 (2.04)	95 (2.57)
Pathological margin status, *n* (%)							
pR0	20 955 (95.62)	2182 (95.70)	4860 (95.80)	2121 (95.11)	1122 (96.31)	7177 (96.17)	3493 (94.33)
pR1	198 (0.90)	26 (1.14)	41 (0.81)	25 (1.12)	4 (0.34)	51 (0.68)	51 (1.38)
Unknown	761 (3.47)	72 (3.16)	172 (3.39)	84 (3.77)	39 (3.35)	235 (3.15)	159 (4.29)
Postoperative chemotherapy, *n* (%)							
Absent	13 187 (60.18)	1376 (60.35)	3184 (62.76)	1364 (61.17)	701 (60.17)	4428 (59.33)	2134 (57.63)
Present	4208 (19.20)	413 (18.11)	861 (16.97)	451 (20.22)	228 (19.57)	1482 (19.86)	773 (20.87)
Unknown	4519 (20.62)	491 (21.54)	1028 (20.26)	415 (18.61)	236 (20.26)	1553 (20.81)	796 (21.50)

Abbreviations: IQR, interquartile range; JSCCR, Japanese Society for Cancer of the Colon and Rectum; MIS, minimally invasive surgery.

### 3.2 Incidence of LNM, OS, and TVI for Right‐Sided Colon Cancers

The incidence of LNM and the OS rate were calculated to determine the TVI. In terms of the incidence of LNM (Figure S1), most of the main lymph node metastasis from cecal cancer remained within #203 (#203, 3.55%; #213, 0.39%; #223, 0.00%) and most of the LNM from ascending colon cancer remained within #203 and #213 (#203, 1.83%; #213, 1.03%; #223, 0.34%). Most of the metastases from transverse colon cancer remained within #223 (#203, 0.31%; #213, 0.31%; #223, 3.41%). The 5‐year OS rate was 58.76% in patients who had LNM from cecal cancer in #203, 59.00% and 62.55% in those with LNM from ascending colon cancer in #203 and #213, respectively, and 56.96% in those with LNM from transverse colon cancer in #223 (Figure S2).

**FIGURE 3:**
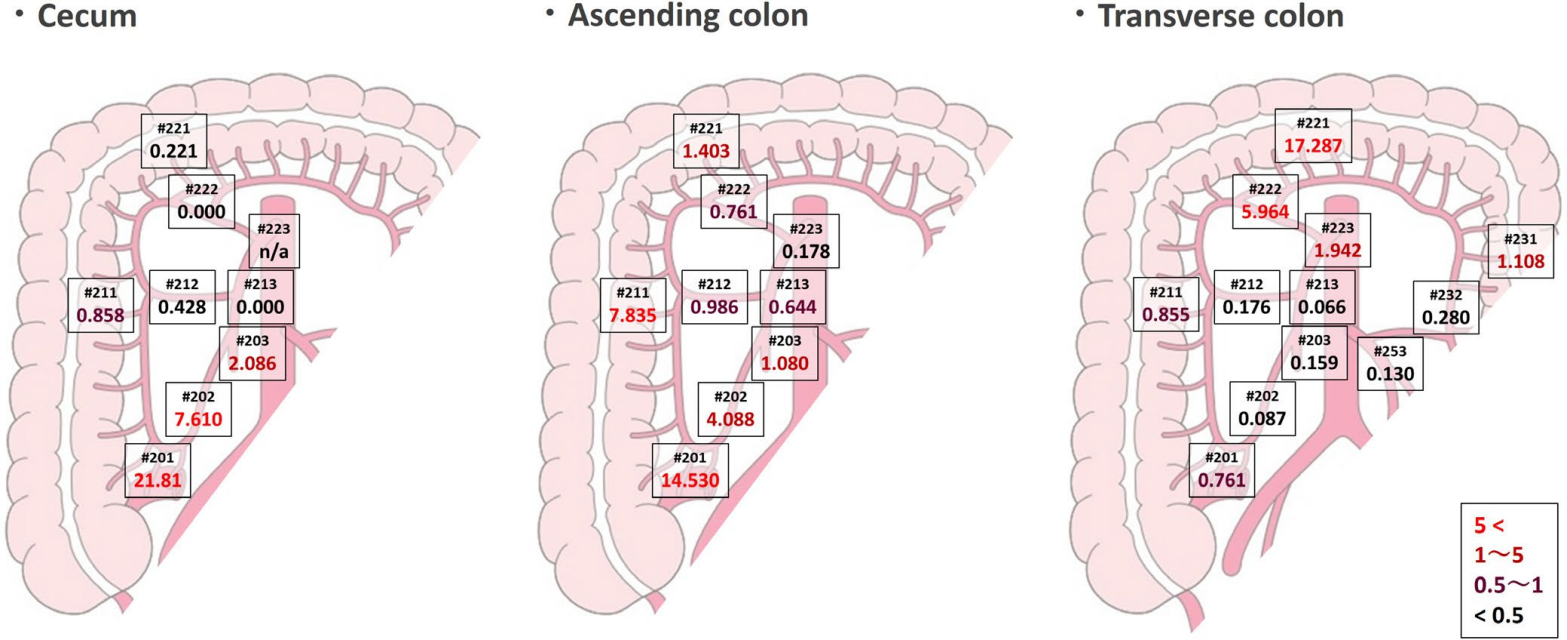
Therapeutic value index of right‐sided colon cancers by tumor location.

Figure 3 shows the TVI calculated for each nodal station when stratified by tumor location. In patients with cecal cancer, the TVI was 2.086 for #203, but only 0.000 for #213 (#223 was not evaluated because there were no LNM). In ascending colon cancer, the TVI was 1.080 and 0.644 for #203 and #213, respectively, but only 0.178 for #223. In transverse colon cancer, the TVI was 1.942 for #223, but only 0.066 and 0.159 for #213 and #203, respectively.

### 3.3 Incidence of LNM, OS, and TVI for Left‐Sided Colon Cancers

The main lymph node metastasis rate from descending colon cancer was 0.86% and 0.00% in #253 and #223, respectively (Figure S3). The metastasis rate was 1.90% at #253 in patients with sigmoid colon cancer and 2.11% at #253 in those with rectosigmoid cancer. The 5‐year OS rate was 25.00% in patients who had LNM from descending colon cancer in #253, 61.69% in those with LNM from sigmoid colon cancer at #253, and 54.76% in those who had LNM from rectosigmoid cancer at #253 (Figure S4).

**FIGURE 4:**
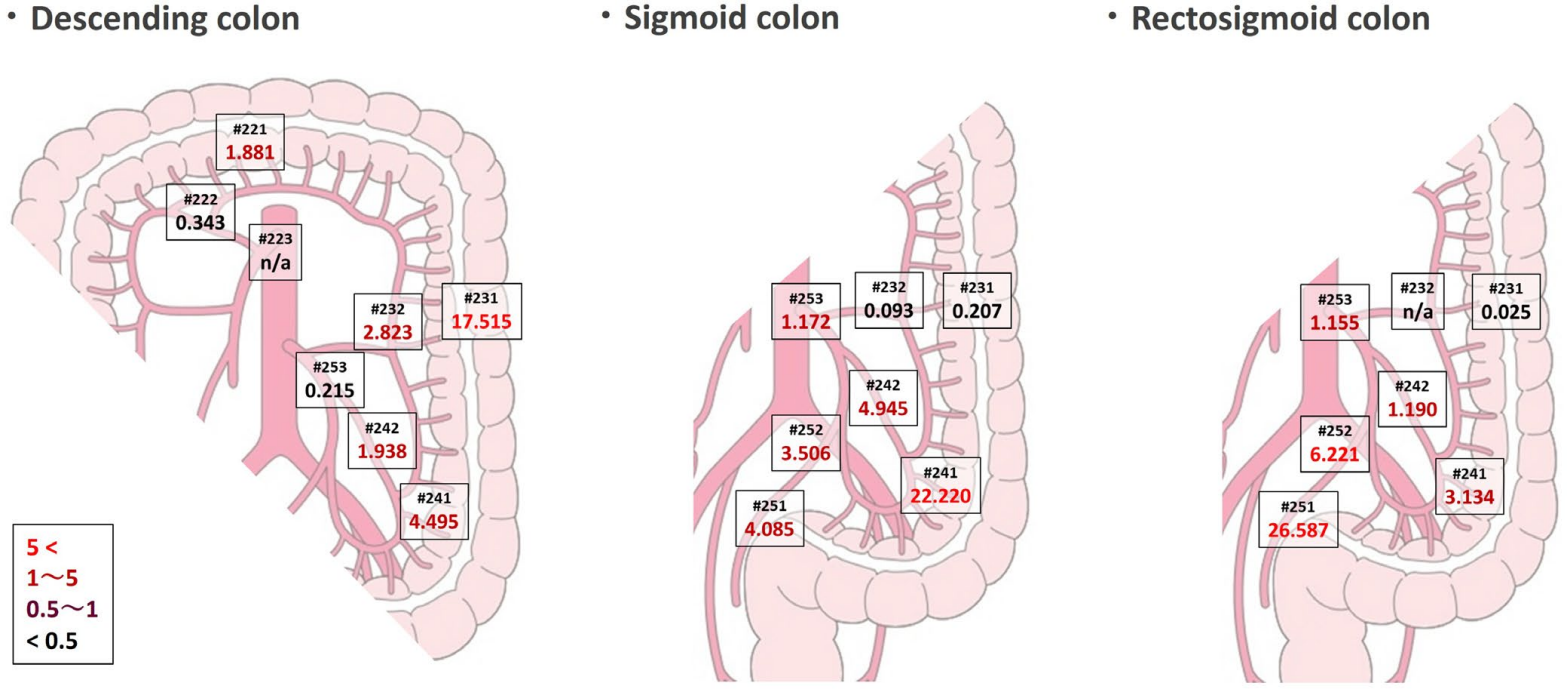
Therapeutic value index of left‐sided colon cancers by tumor location.

The TVI calculated for each nodal station is shown stratified by tumor location in Figure 4. In descending colon cancer, the TVI for #253 was 0.215 (#223 was not evaluated because there were no LNM). The TVI was 1.172 for #253 in sigmoid colon cancer and 1.155 for #253 in rectosigmoid cancer.

## 4 Discussion

This study is the largest to date to calculate the TVI for each lymph node station for colon cancer surgery by tumor location. Most of the main lymph node metastasis remained at the root of the feeding artery (cecum, ascending colon–ileocolic artery, transverse colon–middle colic artery, sigmoid colon, and rectosigmoid–inferior mesenteric artery). The incidence of LNM in these stations was in the range of 1.83%–3.55%, and the 5‐year OS rate in patients who had LNM in these stations was 54.76%–61.69%, resulting in the TVI being in the range of 1.080–2.086.

The TVI is a relative measure that can be used to assess the efficacy of lymph node dissection in each lymph node station and decreases when the incidence of LNM or survival rate after lymph node dissection is low, which means the efficacy of lymph node dissection is limited. Meanwhile, the TVI increases when the incidence of LNM and survival rate after lymph node dissection is high, indicating that lymph node dissection is effective. The TVI has recently been used to investigate the optimal extent of lymph node dissection according to tumor location, surgical procedure, or tumor stage in several types of cancer [16, 17, 18, 19]. The results of some of these studies are reflected in the treatment guidelines for gastric and esophageal cancer in Japan [20, 21, 22].

The TVI itself is a relative measure so that the weight of the index itself varies depending on the patient and disease setting. As a reference, a previous systematic review that investigated para‐aortic lymph node (PALN) metastasis, which is categorized as M1 in both the AJCC [23] and JSCCR classifications, reported an incidence of simultaneous PALN metastasis of 1.3%–1.8% and a 5‐year OS rate after curative resection of simultaneous PALN metastasis of 22.7%–33.9% [24]; accordingly, we estimated the TVI for PALN metastasis from advanced CRC to be in the range of 0.295–0.576. Therefore, along each feeding artery may be higher than that for PALN. Furthermore, the TVI for each of the main lymph nodes was lower than that for the pericolic and intermediate nodes. These results highlight the significance of the JSCCR N3 classification, which is neither N1–N2 nor PALN metastasis.

The present study also provides some important insights regarding the extent of lymph node dissection for non‐metastatic colon cancers. One is the optimal extent of dissection of the main lymph nodes for right‐sided colon cancer. A multicenter prospective observational study from Japan found a low incidence of metastasis from cecal cancer at #213 or #223 and a low incidence of metastasis from transverse colon cancer at #203 [25]. The results of that study are in line with those of the present study, which included a larger number of patients. Therefore, in terms of the borderline of N3 and M1 (LYM) in the JSCCR classification, it seems preferable to define N3 as “the main lymph nodes along the feeding artery” and M1 as “the lymph nodes beyond N3” in patients with colon cancer.

Another open question is the significance of #253 dissection in descending colon cancer. Notably, we observed a low incidence of #253 metastasis, poor survival of patients with metastasis in this station, and a low TVI in descending colon cancer. These results differ from those in the sigmoid colon and rectosigmoid cancer. In 2023, Watanabe et al. mapped LNM for splenic flexural colon cancer and reported that 3.7% of patients had a main lymph node metastasis along the accessory middle colic artery (#223‐acc) [26]. Unfortunately, the lymph node status of #223‐acc has not been recorded in the present database because its JSCCR classification has not yet been defined. These missing data make it difficult to discuss further the optimal extent of dissection of the main lymph nodes for descending colon cancer at this stage; therefore, further research is needed to optimize the extent of dissection in patients with descending colon cancer.

One of our challenges was to make it compatible with the comparability of TVI between each nodal station and the accuracy of the incidence of LNM. In the present study, we calculated TVI based on patients with only D3 lymph node dissection for all nodal stations. This policy was to maintain the comparability between the TVI of each station. Meanwhile, the concern in this policy remains in the overestimation of TVI in pericolic or intermediate lymph nodes due to the exclusion of those with D2 dissection. However, our data still provide valuable insights regarding lymph node dissection of the main lymph node along the feeding artery.

The present study has some limitations, the main one being related to the properties of a real‐world database. About a half of all patients with surgical stage I–III colon cancer during the study period were not eligible for analysis due to inadequate lymph node dissection or missing information targeted for analysis. Nevertheless, we were able to analyze over 20 000 patients, including 600 with main lymph node metastasis, which is the largest number of patients ever enrolled in research on this topic. In addition, the JSCCR database collected information on surgical staging but not on clinical staging during the study period. However, the lack of data on clinical staging did not affect the evaluation of TVI itself. Furthermore, the indications for D3 dissection and its extent depended on the policy of each institution, but all patients included in this database were treated at JSCCR institutions that are dedicated facilities for the treatment of CRC according to the JSCCR guidelines. Meanwhile, as described above, the JSCCR database lacked detailed information about the location of the transverse and descending colon, which hindered our ability to engage in a more thorough discussion regarding the optimal extent of lymph node dissection for transverse and descending colon cancer.

## 5 Conclusions

In this study, dissection of the main lymph nodes along the feeding artery had some degree of therapeutic value for non‐metastatic colon cancers. If this is clearly stated in the JSCCR classification, then it would be desirable to define N3 as “the main lymph nodes along the feeding artery.” Meanwhile, the significance of dissection at #253 for descending colon cancer is still unknown and requires further investigation.

## Author Contributions

**Akira Ouchi:** conceptualization, data curation, formal analysis, writing – original draft. **Kozo Kataoka:** conceptualization, data curation, writing – review and editing. **Eiji Shinto:** conceptualization, writing – review and editing. **Takashi Akiyoshi:** conceptualization, writing – review and editing. **Takefumi Yoshida:** conceptualization, writing – review and editing. **Yasuyuki Takamizawa:** conceptualization, writing – review and editing. **Yukihide Kanemitsu:** conceptualization, supervision, writing – review and editing. **Hirotoshi Kobayashi:** conceptualization, data curation, writing – review and editing. **Yoichi Ajioka:** conceptualization, supervision, writing – review and editing.

## Ethics Statement

Approval of the research protocol by an institutional reviewer board: The independent ethics committees of the Hyogo Medical University (202407‐258) approved the present study as a central institutional review board, and the independent ethics committees of the Aichi Cancer Center executed permission for the present study.

## Consent

Individual patient consent was not required because no identifiable patient data were used.

## Conflicts of Interest

The authors declare no conflicts of interest.

## Supporting information


**Figure S1.** Incidence of lymph node metastasis from right‐sided colon cancers by tumor location.

**Figure S2.** Five‐year overall survival in patients with positive lymph node metastasis from right‐sided colon cancers by tumor location.

**Figure S3.** Incidence of lymph node metastasis from left‐sided colon cancers by tumor location.

**Figure S4.** Five‐year overall survival in patients with positive lymph node metastasis from left‐sided colon cancers by tumor location.

## Data Availability

The data that support the findings of this study are available on request from the corresponding author. The data are not publicly available due to privacy or ethical restrictions.
