# Supplementary material for: Optimal Extent of Lymph Node Dissection for Non‐Metastatic Colon Cancer by Tumor Location: Evaluation of the Therapeutic Value Index for Each Lymph Node Station
Source: Ann Gastroenterol Surg. 2025 Apr 21;9(5):1008–16. doi: 10.1002/ags3.70023 (PMC12414605; doi:10.1002/ags3.70023)
Supplement: Supplementary file 3 — Figure S3. Incidence of lymph node metastasis from left‐sided colon cancers by tumor location. [file AGS3-9-1008-s003.pptx]

## Slide 1
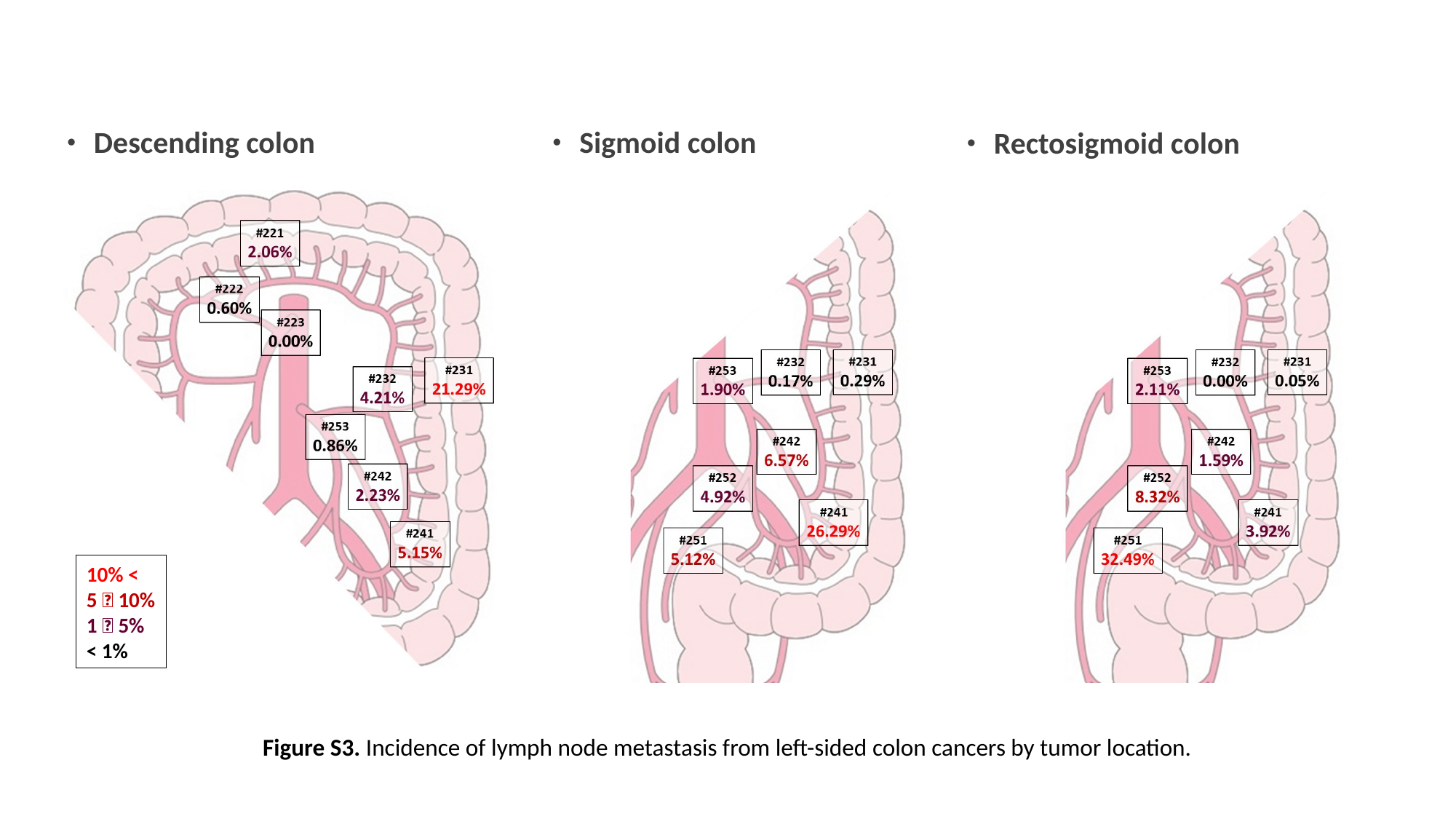

・Sigmoid colon
・Descending colon
・Rectosigmoid colon
10% <
5～10%
1～5%
< 1%
Figure S3. Incidence of lymph node metastasis from left-sided colon cancers by tumor location.
