# Supplementary material for: Optimal Extent of Lymph Node Dissection for Non‐Metastatic Colon Cancer by Tumor Location: Evaluation of the Therapeutic Value Index for Each Lymph Node Station
Source: Ann Gastroenterol Surg. 2025 Apr 21;9(5):1008–16. doi: 10.1002/ags3.70023 (PMC12414605; doi:10.1002/ags3.70023)
Supplement: Supplementary file 4 — Figure S4. Five‐year overall survival in patients with positive lymph node metastasis from left‐sided colon cancers by tumor location. [file AGS3-9-1008-s001.pptx]

## Slide 1
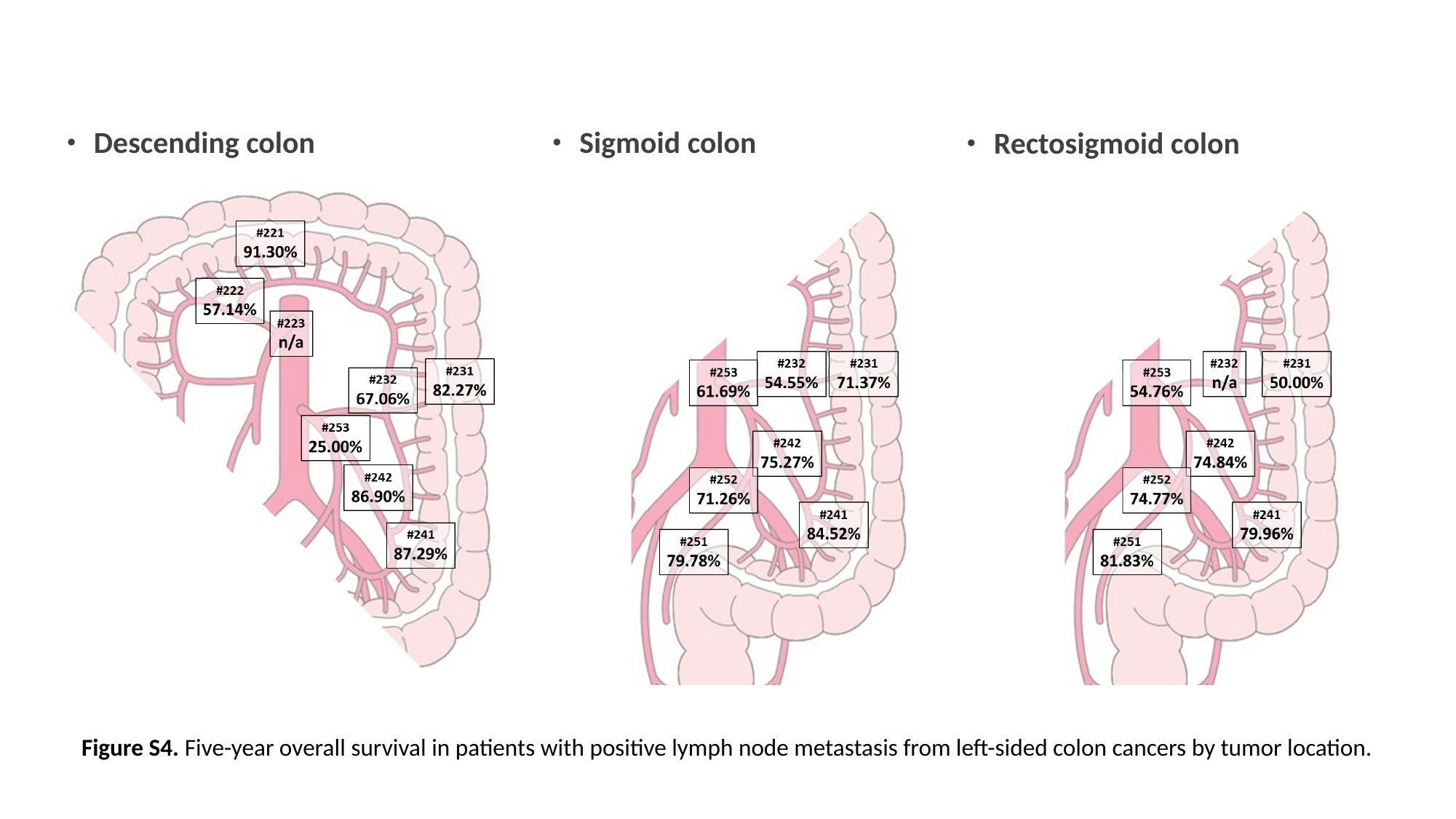

・Sigmoid colon
・Descending colon
・Rectosigmoid colon
Figure S4. Five-year overall survival in patients with positive lymph node metastasis from left-sided colon cancers by tumor location.
